# Is the mosquito the problem? Aedes aegypti and urban arboviruses - contradictions and reflections

**DOI:** 10.1590/S2237-96222023000200024

**Published:** 2023-08-18

**Authors:** Denise Valle, Raquel Aguiar

**Affiliations:** 1 Instituto Oswaldo Cruz, Rio de Janeiro, RJ, Brazil

With the end of the COVID-19 pandemic, the Aedes aegypti vector has returned to the scene, as have the arboviruses it transmits, and along with them we frequently hear the complaint “Why do we still have to live with this mosquito?”

Several aspects contribute to making diagnosis of this situation - and, therefore, overcoming it - confusing, difficult or even unattainable. With the aim of understanding the entirety of this context, we have listed ten points, some either partially overlapping or with contradictory narratives. These points have been perceived over more than two decades of work with vector surveillance and control, from the perspective of biology, and in interaction with health workers and managers, researchers, communicators and other professionals committed to the subject.

## 1. Health versus absence of disease

“Health is often left on its own in this story, as if the problem were only its responsibility”.^1^ This outpouring of the then coordinator of the National Dengue Control Program, during a dengue epidemic in Brazil, reveals the disarticulation between the different sectors of the government. In part, this expresses the reduction of the concept of health to “absence of disease”.

As such, it is important to recall the Ottawa Charter (1986)^2^: “health promotion is not just the responsibility of the health sector, but goes beyond healthy life-styles to well-being. Health is, therefore, seen as a resource for everyday life, not the objective of living’’.

## 2. Taking care with diagnosis: technical solutions versus political and/or structural issues

Since 1986, when the dengue virus settled for good in Brazil, vector control actions have been intensified. Currently, strategies are being multiplied and are generally of a technical nature: in addition to insecticides, approaches such as disseminating stations, sterile males or strains of A. aegypti containing the endosymbiotic bacterium Wolbachia are in focus.^3^ These are technical solutions, with focusing on reducing arbovirus-transmitting mosquito populations. Notwithstanding, A. aegypti has spread throughout approximately 90% of Brazil’s municipalities.^4^

The importance of diagnosis is imperative. How can inequality in the occupation of urban spaces, access to drinking water, garbage collection and sanitation be neglected, among other aspects directly linked to greater risk of exposure to infestation by A. aegypti?^5^ These are manifestations of social, structural and economic vulnerability in Brazil. It is also worth highlighting the emphasis, often accompanied by political bias and welfarist content, placed on certain new “solutions”, presented as the “magic bullet” of the moment.^6^

## 3. A domestic mosquito (public versus private)

Aedes aegypti “likes shade and fresh water” and has adapted itself to living alongside humankind. Although it is synanthropic, it is sometimes treated as being “domestic”. In general, 80% of its breeding sites are indoors or outdoors close to buildings, this being a situation that reiterates the importance of periodic checking for A. aegypti in people’s homes.^3^

In Brazil, there are tens of thousands of endemic disease control health workers (agentes de combate às endemias - ACEs), responsible for home visits, an activity that challenges the concepts of “public” and “private”: they are “public” agents inspecting “private” environments. This ambiguity underlies two distinct behaviors, both of which have the potential impact of increasing infestation levels: (i) limited ACE access - this is widespread and restricts visits to more than 50% of homes; and (ii) adoption of negligent behavior in relation to the domestic space, based on argument that “it’s the job of the ACEs”.

## 4. The know-do gap

This term refers to the gap between having knowledge of a problem and doing something to solve it. A classic example is the “physician who smokes”. Overcoming the know-do gap requires a change in an individual’s behavior, which is no mean feat. Collectively, changes of this type are encouraged by awareness-raising campaigns, effective communication strategies or even adoption of regulations associated with penalties. Using seat belts while driving is an example of this, as it is monitored in public environment.

With regard to Aedes, the practice of routinely eliminating breeding sites, the most effective form of prevention with regard to the domestic environment, has an additional contradiction, as it requires a change in the conduct of the community in private environments, generally inaccessible to screening by the public sector. The know-do gap also involves perception of risk: in the case of seat belts, for those who have not experienced an accident, being fined is the “risk”; in the case of Aedes, an arbovirus can mean a “limited” and transient risk - and is therefore easily forgotten.

## 5. The 10/90 gap

The original expression indicates that just 10% of resources for health research relate to conditions that affect 90% of the diseases burden. These are the neglected diseases, or populations.^6^ The concept, however, can be extrapolated to other contexts. With regard to Aedes, it could be said that 90% of control actions reach only 10% of mosquito populations; or even that 90% of resources are used to solve peripheral aspects of the issue which have greater visibility.

## 6. Paying attention to diagnosis: technical solutions versus false sense of security

Three peculiarities of the vector’s biology corroborate the relevance of periodic prevention measures: (i) Aedes spreads its eggs in many breeding sites; (ii) A. aegypti can become adults as little as 7-10 days after the eggs are hatched; and (iii) spraying with insecticides only has an effect on mosquitoes that fly through the spray. It is common for ACE visits, every two months, or one-off insecticide spraying to kill adults^7^ to be perceived with relief, as the “right” solution(s). Considering the limited effectiveness of insecticides and the habits of A. aegypti, this sense of security may result in reckless relaxation of prevention in the domestic environment. 

## 7. Control versus chemical control

“Mosquito control”, as popularly conceived, is practically synonymous with “use of insecticides”; or even ultra-low volume (ULV) insecticide spraying. The confusion between “control” and “chemical control of the vector”, or even “chemical control of the adult vector”, is even institutionalized in some situations.

There are two ideas underlying this misconception. The first relates to the perspective of a magical solution to the problem - attributed in a reductionist way as being the mosquito. The second consists of welfarism being considered natural, as opposed to taking an attitude that is participatory and citizen.

In its origins, the term “vector control” means reducing infestation with approaches that reach, indiscriminately, all components of the vector population - in this scenario, mechanical control is highlighted. The effect of chemical control is not homogeneous across populations. Insecticides select naturally resistant vectors, increasing their frequency in the population. When the frequency of resistant vectors is very high, insecticides lose their effect.^3^ ^),(^ ^8^ Spraying is a further exacerbation of this misconception. Its impact is even more limited as it depends on the insecticide’s ability to reach vectors that fly.^8^

However, when insecticides are used as a complement to mechanical control, there is “room” for vectors with varied profiles to be replaced, whether or not they are resistant to insecticides.

## 8. A metaphor of war: public enemy versus “pet” mosquito

Aedes aegypti is a public enemy. This metaphor of war^9^ refers refers to an external threat and to the unsustainable posture of exceptionality.

Here, it is appropriate to say that the “enemy” is “friendly fire”. Negligence with domestic spaces and spaces in common, and neglect with actions that mitigate inequalities, such as basic sanitation and garbage collection, contribute to the presence of the vector. The contradiction between the discourse, which combats Aedes, and the practice, which gives the vector the status of a pet, with the guarantee of multiple breeding sites, is evident.

## 9. Arbovirus epidemic or information epidemic?

Media and science have different and sometimes conflicting dynamics and logics. The media conveys news, while science tests hypotheses. The media tends to scale up conflicts, while science investigates solutions.

Two recent global health emergencies have had antagonistic effects on Aedes surveillance. In 2015, the identification of the relationship between Zika virus and congenital syndrome in newborns caused panic and intensified the search for vector control tools. In 2020, COVID-19 practically made arboviruses invisible, even interrupting entomological surveillance, based on home visits by ACEs.^10^

Both situations reveal the need for articulation between science and media, as well as health service management: (i) science, notably biomedical science, requires recognition of the relevance of structural determinants and the limitation of solutions with exclusive focus on the vector; (ii) in the case of the media, the challenge is conceptual, it consists of turning awareness of behaviors that should be everyday behaviors into news; and (iii) health service management needs to be less vertical and not to impose communication so much, but rather value solutions that favor citizenship and minimize inequalities, to the detriment of “magic bullets”.

## 10. Vector control as a business opportunity

Today there are more people living with dengue than dying from it. Or “living thanks to Aedes”.

Public health management has a consolidated structure reaching every municipality in the Brazil. Private companies proliferate, as a rule they are chemical industries. Biomedical research multiplies innovative and technological solutions. The focus is always on the mosquito, recognized as a vulnerable link in the transmission chain. This results in all public structures, private companies and technological initiatives being maintained.

In practice, A. aegypti is the major employer, maintainer and articulator of a thriving “mixed economy” conglomerate, with benefits for many of those involved. On the other hand, the human drama of those affected by arboviruses continues. This equation is unjust.

Good medication depends on correct diagnosis. The question is: to what extent does diagnosis mean each individual position being kept in place on the chessboard (or, in the “breeding sites”) of opportunities?
